# Reprogramming DNA methylation in the mammalian life cycle: building and breaking epigenetic barriers

**DOI:** 10.1098/rstb.2011.0330

**Published:** 2013-01-05

**Authors:** Stefanie Seisenberger, Julian R. Peat, Timothy A. Hore, Fátima Santos, Wendy Dean, Wolf Reik

**Affiliations:** 1 Epigenetics Programme, The Babraham Institute, Cambridge CB22 3AT, UK; 2 Centre for Trophoblast Research, University of Cambridge, Cambridge CB2 3EG, UK; 3 Wellcome Trust Sanger Institute, Cambridge CB10 1SA, UK

**Keywords:** DNA methylation, germline, reprogramming, development, hydroxymethylation, epigenetics

## Abstract

In mammalian development, epigenetic modifications, including DNA methylation patterns, play a crucial role in defining cell fate but also represent epigenetic barriers that restrict developmental potential. At two points in the life cycle, DNA methylation marks are reprogrammed on a global scale, concomitant with restoration of developmental potency. DNA methylation patterns are subsequently re-established with the commitment towards a distinct cell fate. This reprogramming of DNA methylation takes place firstly on fertilization in the zygote, and secondly in primordial germ cells (PGCs), which are the direct progenitors of sperm or oocyte. In each reprogramming window, a unique set of mechanisms regulates DNA methylation erasure and re-establishment. Recent advances have uncovered roles for the TET3 hydroxylase and passive demethylation, together with base excision repair (BER) and the elongator complex, in methylation erasure from the zygote. Deamination by AID, BER and passive demethylation have been implicated in reprogramming in PGCs, but the process in its entirety is still poorly understood. In this review, we discuss the dynamics of DNA methylation reprogramming in PGCs and the zygote, the mechanisms involved and the biological significance of these events. Advances in our understanding of such natural epigenetic reprogramming are beginning to aid enhancement of experimental reprogramming in which the role of potential mechanisms can be investigated *in vitro*. Conversely, insights into *in vitro* reprogramming techniques may aid our understanding of epigenetic reprogramming in the germline and supply important clues in reprogramming for therapies in regenerative medicine.

## 1. Introduction

Mammalian development begins with the totipotent zygote, which has the developmental potential to generate an entire organism. This totipotent state is not defined by a unique genetic complement—almost without exception, all cells descended from the zygote share its exact DNA sequence despite having a restricted developmental capacity. Thus, ‘epigenetic’ features (or lack thereof) must define the developmental potency of the zygote and promote canalization towards a distinct cell fate in future cell generations [1]. Histone tail modifications and methylation of the fifth carbon of the cytosine base (5mC) in DNA itself are perhaps the best-studied epigenetic modifications in mammals, although the epigenetic lexicon is rapidly expanding to include other interdependent phenomena such as non-coding RNAs and higher-order chromatin organization. Presumably, epigenetic marks, including 5mC, provide an epigenetic barrier that reduces developmental potential while promoting distinct cellular identity. This identity is stably inherited from one cell division to the next through the DNA methylation maintenance machinery. The key players are nuclear protein 95 (*NP95* or *Uhrf1*), which recognizes hemimethylated DNA at replication foci [2,3], and DNA methyltransferase 1 (*Dnmt1*), which then copies DNA methylation marks from the parental strand onto the newly synthesized daughter strand [4]; the extent to which other epigenetic marks are mitotically heritable is under investigation.

**Figure 1.**
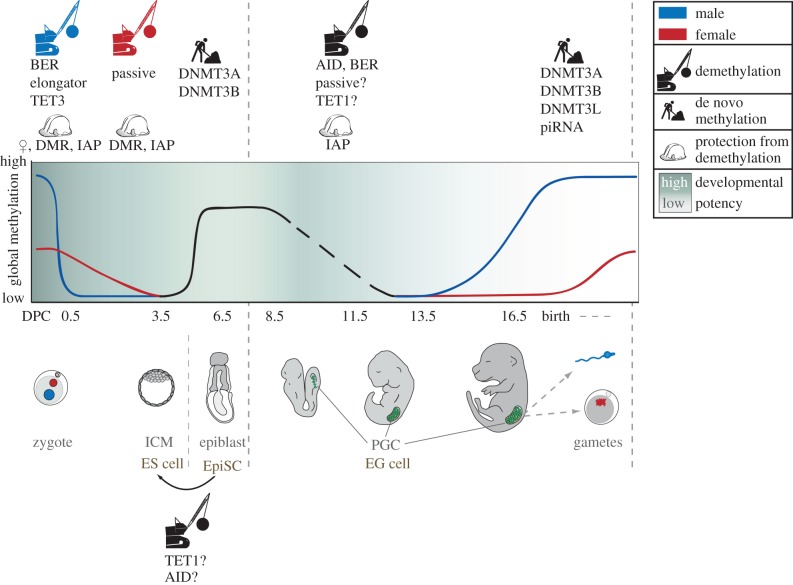
DNA methylation reprogramming in the mammalian life cycle. DNA methylation marks represent an epigenetic barrier in mammalian development that is demolished when developmental potency has to be restored and subsequently re-built with the commitment to a particular cell fate. This first occurs following fertilization, when the DNA methylation marks of the parental gametes are erased in two waves of demethylation. In the first wave, the paternal pronucleus (shown in blue) undergoes rapid demethylation in the zygote, which is followed by a passive loss of DNA methylation marks in the maternal genome (shown in red) over the subsequent cell divisions. Re-establishment of DNA methylation marks commences in the ICM of the developing embryo, which forms an epigenetic barrier (dashed line) in the developmentally more restricted epiblast. PGCs (shown in green) inherit the epigenetic signature from the epiblast, and DNA methylation is again erased on a global scale concomitant with the restoration of developmental potency. Note that DNA methylation at DMRs of imprinted genes become reset in PGCs but are protected from reprogramming in the early embryo. With further development into fully specialized gametes, DNA methylation marks are re-established and developmental potency is restricted. This epigenetic barrier (dashed line) will be demolished once more in the zygote of the next generation as part of the continuous cycle of DNA methylation reprogramming.

Transitions in cell fate and restoration of developmental potency are closely associated with some form of epigenetic reprogramming. Indeed, in the germline, there are two genome-wide DNA demethylation events coincident with major developmental milestones (figure 1): (i) immediately following fertilization in the zygote, and (ii) during the establishment of the primordial germ cells (PGCs), which are the direct progenitors of sperm and oocytes. It is thought that these dramatic changes in epigenetic status allow the zygote to erase the epigenetic signature inherited from the gametes (with the notable exception of parental imprints) and thereby regain developmental totipotency. Likewise, epigenetic reprogramming of PGCs is associated with restoration of developmental potential and the erasure of parental imprints. PGCs derive from the epiblast—a tissue with high developmental capacity, but also one that is characteristically directed towards somatic lineages and requires significant reprogramming to restore the germline.

**Figure 2.**
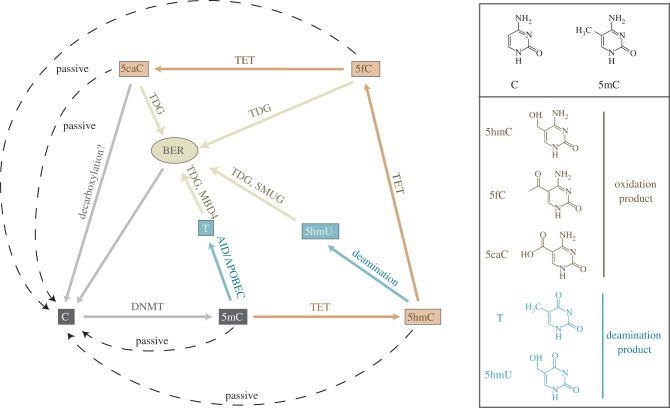
Pathways for removal of DNA methylation. Cytosine (C) is methylated at the 5′ carbon position by DNMT enzymes to generate 5-methylcytosine (5mC). This can be lost passively owing to a lack of maintenance at DNA replication (dashed line), or actively processed by enzymatic activity. 5mC can be deaminated to thymine (T) by the AID/APOBEC deaminases (blue), or oxidized to 5-hydroxymethylcytosine (5hmC) by the TET enzyme family (brown). 5hmC itself may be deaminated to 5-hydroxymethyluracil (5hmC), or further oxidized by TET activity to 5-formylcytosine (5fC) and 5-carboxylcytosine (5caC). The T, 5hmU, 5fC and 5caC derivatives can be excised by glycosylases (beige) such as TDG, single strand-selective monofunctional uracil DNA glycosylase 1 (SMUG1) and methyl-CpG-binding domain protein 4 (MBD4) to initiate the BER pathway resulting in their replacement with unmodified C. Alternatively, 5fC and 5caC can be lost passively through lack of maintenance; 5caC may also be converted to C by a decarboxylation reaction. For clarity, demethylation catalysed by the elongator complex is not shown.

Recent advances have begun to elucidate how such dramatic demethylation in the zygote and PGCs is orchestrated, but a clear picture of the mechanistic details of this reprogramming and its consequences has not yet emerged. DNA methylation can be lost either through ‘passive’ dilution owing to a lack of maintenance at replication, or by ‘active’ enzyme-catalysed removal of 5mC from the DNA (figure 2). A direct DNA demethylase that is capable of cleaving the carbon–carbon bond between the methyl-group and the deoxyribose of the cytosine (C) has not been identified in mammals, but recent work has explored indirect demethylation pathways that involve deamination or oxidation of 5mC potentially coupled with base excision repair (BER; figure 2). Deamination of 5mC and C by the deaminases AID and APOBEC1 can initiate BER pathways, including potentially the glycosylases TDG and MBD4 as well as the DNA damage response protein GADD45 [5]. Oxidation of 5mC to 5-hydroxymethylcytosine (5hmC) and further to 5-formylcytosine (5fC) and 5-carboxycytosine (5caC) can have two consequences: it can abolish the generally repressive effect of the original 5mC and it can be replaced by unmodified cytosine through various routes potentially, including DNA replication, deamination and BER [6]. Research using new mouse models targeting these putative demethylation pathways has provided evidence for their involvement in germline reprogramming [7–9]. In addition, cell-culture paradigms representing different stages of the germline have recently been developed, and study of how these models—which vary in their developmental potency—may be interconverted has proved fruitful in uncovering the significance of DNA methylation reprogramming. Here, we review novel insights into how DNA methylation is reprogrammed in the mouse germline and speculate on its purpose.

## 2. DNA methylation reprogramming in primordial germ cells

PGCs first arise around E7.25 in the epiblast of the developing embryo [10] and, at these early stages, seem to inherit the epigenetic traits that are present in the cells of the epiblast at this time, including significant levels of global DNA methylation [11,12]. As a consequence, PGCs need to reprogramme this inherited somatic epigenetic pattern into that of germ cells that have the epigenetic potential to give rise to the gametes, with the capacity to form the totipotent zygote in the next generation (figure 1). Epigenetic reprogramming in PGCs is a dramatic undertaking that leads to the resetting of most DNA methylation marks—exceptions include the most active retrotransposons, those with mutagenic potential, such as intracisternal A particles (IAPs), that resist the global wave of demethylation [13–15]. Sequences that resist reprogramming may potentially act as carriers of epigenetic information across generations, leading to transgenerational epigenetic inheritance. Epigenetic reprogramming in PGCs also entails remodelling of the chromatin structure, potentially vast changes in the transcriptional landscape and the resetting of imprint DNA methylation marks [11,13,16,17]. The latter have been the subject of intensive studies since the discovery, almost 20 years ago, that the maternal and paternal copy of some genes are differentially marked by DNA methylation, leading to parent-of-origin-specific expression [18]. We now know that these imprinted genes play important roles in regulating growth in embryonic and postnatal development, as well as behaviour [19]. It is crucial for the development of the next generation that the parental imprints are erased in PGCs and that new imprints are established that reflect the gender of the embryo. These imprints are then maintained in the gametes derived from the PGCs and will contribute to the epigenome of the zygote.

The resetting of the epigenetic signature inherited from the epiblast re-establishes developmental potency in PGCs. Indeed, pluripotency markers such as *Oct4*, *Stella*, *Nanog* and alkaline phosphatase become transcriptionally active in PGCs [20–23]. In addition, pluripotent embryonic germ (EG) cells can be derived from various stages of developing PGCs, which show highly similar characteristics to ES cells and can also contribute to chimaeras when injected into mouse blastocysts [24–27]. These EG cells appear to be even more potent in their reprogramming potential than ES cells—in somatic cell reprogramming, only EG cells can erase imprints from their somatic fusion partners [28,29]. Intriguingly, the re-gained pluripotent state in PGCs is only transient as the pluripotency network becomes transcriptionally downregulated thereafter both in male and in female PGCs by E16.5 (S. Seisenberger *et al.* 2012, unpublished data). It is unclear at this point what the mechanistic function of the activity of the pluripotency network in PGCs might be, and why this activation is only transient.

Investigations into the mechanisms of global DNA methylation erasure in PGCs have largely focused on the period between E11.5 and E13.5, as the classic model describes global DNA methylation erasure occurring concomitant with imprint erasure from E11.5 [13,30]. This model implies that DNA methylation erasure is at least in part an active rather than a passive process, as this period is considered too short to allow for passive loss of DNA methylation marks over several cell divisions.

Recent advances have identified a number of proteins that promote active demethylation of specific loci under certain conditions. One of these proteins is activation-induced deaminase (*Aid* or *Aicda*), which contributes to demethylation of the *Oct4* and *Nanog* promoters in somatic cell reprogramming [31]. *In vitro* AID can deaminate 5mC to thymine (as well as C to U) [32], which can then be recognized by the thymine DNA glycosylases (TDG and MBD4) as a potentially mutagenic T–G mismatch and excised using the BER pathway [5] (figure 2). Replacement with an unmethylated cytosine prior to or at replication results effectively in demethylation. AID is the only protein for which involvement in global erasure of DNA methylation marks in PGCs has been demonstrated [7]. However, the epigenetic phenotype upon *Aid* depletion in PGCs is moderate, which strongly suggests the presence of additional demethylation mechanisms that either compensate for the lack of AID activity or act on different sequence targets.

Oxidation of 5mC to 5-hydroxymethylcytosine (5hmC) by members of the ten-eleven-translocation (*Tet*) family is another attractive candidate for global methylation erasure, as it allows for rapid removal of 5mC potentially without a mutagenic intermediate (figure 2). The resulting 5hmC can be lost passively over subsequent cell divisions through a lack of maintenance at DNA replication, although we note that NP95—part of the maintenance methylation machinery—has the capacity to bind 5hmC [33]. In addition, recent studies have provided evidence that conversion of 5mC to 5hmC can result in further processing to unmodified cytosine via the BER pathway. In mouse brain, this appears to occur via an initial deamination of 5hmC by the Aid/Apobec family of deaminases, and subsequent excision by a range of glycosylases [34]. However, recent biochemical evidence suggests that 5hmC is an unlikely substrate for enzymes of the AID/APOBEC family and further analysis of the molecular mechanims of 5hmC deamination is needed [35,36]. Alternatively, 5hmC can be further oxidized by the TET enzymes to 5fC and 5caC, which can be excised by TDG [37–39] (figure 2). It is currently unclear whether the oxidation products of 5mC are themselves functional modifications, or simply intermediates on the route to unmodified cytosine. Interestingly, depletion of *Tdg* in mouse embryos disrupts promoter methylation and histone architecture at a range of loci resulting in embryonic lethality, and *Tdg*-depletion in PGCs leads to hypermethylation of imprinted genes [8,40]. In addition, there is evidence that BER components such as poly(ADP-ribose)-polymerase 1 (*Parp1*), apurinic/apyrimidinic endonuclease 1 (*Ape1*) and *Tet1* are transcriptionally upregulated in E11.5 PGCs [41]. However, a role for TDG, BER and the TET proteins in global methylation erasure in PGCs has yet to be uncovered.

In contrast to the classical model, there have been reports about DNA methylation erasure starting as early as E8.0 [11], which is in line with the transcriptional down regulation of the DNA methylation machinery prior to this point [16]. Recent molecular evidence now suggests that global erasure of DNA methylation marks may indeed begin as early as E8.5 and that imprint DNA methylation marks, from which the classical model was derived, may have different erasure kinetics to the rest of the genome [15]. Increasing evidence pointing towards an earlier demethylation phase for the bulk of the genome may necessitate a shift in experimental focus towards earlier time points in PGC development. Furthermore, if global erasure in PGCs begins early, possibly from E8.0, the window for DNA demethylation extends over a much longer period and could thus comprise a greater number of cell divisions. This places the possibility of passive demethylation back on the table. In line with these results, it has been hypothesized that 5mC—and its oxidized derivatives—could be lost owing to a lack of maintenance over several cell divisions, culminating in the extremely hypomethylated state at E13.5 [42,43].

Following demethylation in early PGCs, the genome must undergo de novo methylation in order to achieve the much higher levels of methylation found in mature gametes (figure 1). Our understanding of methylation re-establishment in PGCs is again patchy and largely derived from the kinetics of methylation establishment at imprinted differentially methylated regions (DMRs) and retrotransposons: de novo methylation in male PGCs takes place several days after erasure is completed, between E14.5 and E16.5, depending on the mouse strain, and continues until the prospermatogonia phase [44–47]. In female germ cells, DNA methylation establishment takes place after birth in the growing oocyte [48–52]. The result of de novo methylation is a DNA methylation pattern reflecting germ cell fate with a set of imprints representative of the sex of the embryo. The establishment of DNA methylation marks, including those of imprinted genes in male and female PGCs, has been shown to require the de novo methyltransferases *Dnmt3a* and *Dnmt3b*, the non-catalytic orthologue *Dnmt3l*, and for some maternal imprints, the histone 3 lysine 4 (H3K4) demethylase KDM1B [47,53–56]. In this pathway, DNMT3L is recruited to unmethylated H3K4 tails and in turn recruits DNMT3A and/or DNMT3B, leading to local de novo methylation of associated sequences [57]. However, it seems that there is some variation in whether DNMT3A or DNMT3B is recruited, and it is clear that there is at least one other mechanism that involves transcription through the DMR to establish DNA methylation marks at imprinted regions [47,58]. The mechanisms for establishment of imprint DNA methylation marks seem to be different from DNA methylation re-establishment at transposable elements, which involves piwi-interacting RNAs (piRNAs) that are mainly expressed in the germline [59]. It thus seems that multiple mechanisms may have evolved to achieve methylation re-establishment in different parts of the genome. Interestingly, the endpoint of global methylation re-establishment seems to be different for male and female germ cells: sperm is heavily methylated with approximately 85 per cent global CG methylation levels, while oocytes are moderately methylated with global methylation levels around 30 per cent [7,52,60].

## 3. DNA methylation reprogramming in the zygote

The DNA methylation patterns established in sperm and oocyte are reprogrammed once more when the two halves of the germline are reunited in the zygote after fertilization (figure 1). The genomes contributed by each parent—independently packaged in separate pronuclei—follow highly distinct paths involving extensive epigenetic remodelling; DNA methylation dynamics is conspicuous in its asymmetry between these pronuclei. The paternal genome is stripped of much of its methylation in a global and active process that appears to occur in two stages—before and coincident with DNA replication—and is complete before the first cell division [61,62]. The maternal genome escapes such comprehensive 5mC loss in the zygote, and is instead passively demethylated over subsequent cleavage divisions owing to the exclusion of the maintenance DNA methyltransferase, DNMT1, from the nucleus [63].

Immunofluorescence with antibodies against 5mC established loss of paternal methylation on a global level [61,64,65]; subsequent bisulphite analysis has shown removal of 5mC from a number of specific loci, including repetitive elements such as Line1 retrotransposons, along with several single copy genes including *Oct4* and *Nanog* [9,14,60,62,64,66–68]. Intriguingly, these molecular analyses also identified paternal sequences that avoid the wave of demethylation; these comprise paternally imprinted genes, IAP retrotransposons (as in PGCs) and heterochromatin in and around centromeres [69]. Successful progression through early cleavage stages may depend on the retention of methylation at these sequences—for the safeguarding of parental imprinting, repression of transposition and chromosomal stability, respectively.

A number of models for active loss of DNA methylation have been proposed [69–71]. Evidence supports the existence of three of these pathways in the zygote: processing through BER, a radical SAM mechanism and enzymatic oxidation of 5mC (described below). It is possible that different pathways may operate sequentially, or in parallel, to form a complex demethylation network.

Components of the BER pathway localize specifically to the paternal pronucleus during the later phase of demethylation, accompanied by the appearance of γH2A.X foci which mark DNA breaks—a hallmark of BER [41,62]. Small molecule inhibition of two BER proteins, PARP1 and APE1, results in increased methylation of the paternal genome both globally and at Line1 elements [41]. While this indicates that a functional BER pathway is required for complete demethylation, further work is required to identify the upstream event (e.g. deamination) that initiates its activity on 5mC.

Using a siRNA knockdown strategy, coupled with live-cell methylation imaging of zygotes, Okada *et al.* [72] identified three components of the elongator complex that are needed to fully demethylate the paternal genome. The elongator complex possesses lysine acetyl transferase activity and appears to perform diverse cellular functions, including the regulation of transcriptional elongation through acetylation of histone H3 [73]. Interestingly, a dominant negative approach revealed that the radical SAM domain, but not the acetyltransferase activity, was required for normal demethylation [72]. While the SAM domain is essential for structural integrity of the complex [74], it is also possible that this domain acts directly to remove 5mC [71]. Should this prove to be its mechanism, this would constitute the first report of a true demethylase in mammals.

Immunofluorescence provides striking evidence for the oxidation of 5mC in the zygote. Concomitant with the loss of methylation signal in the paternal pronucleus, there is a strong increase in antibody staining for 5hmC, as well as the more recently discovered 5fC and 5caC [9,68,75–77]. TET3 is the oxidase responsible: it is highly enriched in the zygote where it appears to bind specifically to paternal chromatin, and its ablation by RNA knockdown or genetic deletion abolishes the generation of 5hmC [9,75]. Crucially, this also precludes complete demethylation, indicating that oxidation is a key pathway for removal of 5mC from paternal DNA. Although these oxidation products have been shown to feed into the BER pathway in brain and ES cells [34,37,38], the enzyme required for this activity, TDG, was not detected in zygotes by immunofluorescence [41]. It is possible that other activities operate in the zygote in the place of TDG, or that oxidized bases are processed independently of BER to generate unmodified cytosine by a decarboxylation reaction [78]. However, immunofluorescence studies indicate that passive loss contributes significantly to demethylation, rather than being processed to unmodified cytosine; a significant amount of 5hmC, 5fC and 5caC are retained in the paternal genome and gradually diluted over cleavage divisions [9,76,77]. It is important to note that these analyses are not quantitative and only assess global patterns—it is possible that different pathways may operate to demethylate specific loci. Conceptually, it is intriguing that the paternal genome should require active oxidation to permit passive demethylation when the maternal genome achieves this without modification of 5mC. This may simply be a consequence of differences in chromatin state between the two pronuclei [1], but alternatively could hint at an unknown functional role for the oxidized bases in the early embryo—perhaps mediated by the binding of proteins that specifically recognize these modifications, such as MBD3 [79].

As bisulphite analysis reads 5hmC as a ‘methylated’ cytosine [80], its global retention appears to be at odds with the bisulphite data that suggested a complete loss of methylation at several loci in the paternal genome. However, the oxidation of some 5hmC to 5fC and 5caC (which are read as ‘unmethylated’ cytosines [37]) along with the parallel operation of non-oxidative BER and mechanisms involving the elongator complex may reconcile these findings. This complexity demonstrates the clear need for the development of molecular techniques that can discriminate the various cytosine modifications to provide quantitative and locus-specific information. Such analysis is likely to uncover considerable variation in the way 5mC is processed at different regions of the genome. In a promising advance, two groups recently reported the development of techniques that provide quantitative base pair-resolution for both 5mC and 5hmC [81,82], although the current requirement for large quantities of sample DNA represents an obstacle for work with zygotes and also PGCs.

Despite exposure to an identical ooplasm, the maternal genome and the paternal sequences described earlier escape active demethylation (figure 1). Intriguingly, the maternal factor Stella (Dppa3 or PGC7) is required for this protection; deletion from the zygote results in demethylation of the maternal genome and paternally imprinted sequences, preventing normal preimplantation development [83,84]. While Stella protein is present in both zygotic pronuclei, its binding is mediated by the H3K9 dimethylation modification—which marks only maternal chromatin and certain paternal imprints to specifically safeguard these regions [85,86]. Mechanistically, this protection is achieved by abrogation of known demethylation pathways: Stella inhibits binding of TET3 to chromatin to prevent oxidation of 5mC [86], as well as suppressing BER component activation in the maternal pronucleus [41]. The interaction of Stella with the BER pathway, as well as other zygotic demethylation machinery such as the elongator complex, requires further analysis; such investigations will also shed light on whether additional factors cooperate with Stella or act independently to shield methylation from processing in the zygote.

## 4. Post-zygotic DNA methylation and developmental potency

During early cell division in a mammalian embryo, daughter cells derived from the zygote inherit a reprogrammed genome with low methylation and are epigenetically largely indistinguishable from each other. The first event that differentiates cells in the embryo occurs at the morula stage; those with a peripheral location are largely destined to become the extraembryonic tissue, while centrally located cells will form the embryo proper [87]. By the blastocyst stage, epigenetic differences are sufficiently obvious between these two lineages to be detected by immunofluorescence [61]: while the outer trophectoderm cells have low levels of DNA methylation, the inner cell mass (ICM) that gives rise to the embryo proper has already undergone some re-establishment of methylation (figure 1). Among those sequences that become methylated in the epiblast, and in this case also silenced, is the *Elf5* gene [88], which is a key determinant of the trophectoderm lineage. In doing so, this epigenetic change provides a stable molecular mechanism that contributes to separating the trophectoderm and the epiblast lineages, an event that has been compared with ‘canalization’ of developmental trajectories within Waddington's model of cellular differentiation [1].

In addition to methylation of *Elf5*, some 500 genes are subject to de novo methylation around the time of implantation [89]. These further methylation changes coincide with another important restriction in developmental potential; while ICM from one blastocyst can be injected into another and successfully contribute to the offspring, similar transplantation of the epiblast beyond E4.5 fails to produce mouse chimaeras [90,91]. This stark developmental restriction is reflected in the *ex vivo* cell-culture model of the epiblast. EpiSCs cultured from E5.5 to 7.5 embryos do not contribute to chimaeras when injected back into blastocysts [92,93], whereas ES cells derived from E2.5 to 4.5 ICM do [93,94]. This difference in pluripotent capacity is in some ways puzzling because EpiSCs, in a similar fashion to ES cells, have the ability to differentiate into all three germ layers, form teratomas and express many markers of pluripotency including *Oct4* [92,93].

Efforts have been made to define the molecular difference between EpiSCs and ES cells that explains their distinct developmental potential. Significantly, EpiSCs lack high expression of key markers of ground state pluripotency, such as *Nanog*, *Esrrb*, *Fbxo15*, *Tcl1*, *Klf2* and *Zfp42* [93,95,96]. These expression differences are more than just indicative of cellular state; simply by overexpressing pluripotency genes such as *Klf4* [96] or *Nanog* [97], it is possible to reprogramme EpiSCs into an induced pluripotent stem cell (iPSC) that is almost indistinguishable from an ES cell. Interestingly, many of the pluripotency genes silenced in EpiSCs and the epiblast are simultaneously subjected to DNA methylation ([89,95] and T. A. Hore *et al.* 2012, unpublished data). During the process of reprogramming EpiSCs to iPSCs, genes such as *Stella* and *Zfp42* undergo demethylation of their promoters in concert with activation of their expression [95], implying that the boundary between ES cells and EpiSCs is, at least in part, epigenetically defined. In support of this concept and its consequences, it has been shown that supplementation of cell-culture medium with 5-azacytidine (a potent inhibitor of DNA methyltransferase activity) can considerably enhance the conversion of EpiSCs to iPSCs, which are highly similar to ES cells in their developmental potential [97].

The TET hydroxylases may also play a significant role in the transition between the bona fide pluripotency of the ICM, and restricted pluripotency in the epiblast. When ES cells are differentiated in culture to form embryoid bodies, *Tet1* is rapidly downregulated [98] alongside many genes that appear to be particularly sensitive to the loss of pluripotency, such as *Esrrb*, *Zfp42*, *Klf2* and *Tcl1* [99]. Strikingly, knockdown of *Tet1* mRNA in ES cells results in reduced expression of these genes and increased levels of DNA methylation in their promoters, implying that they are targets of TET1 protein [100]. Preliminary data from our laboratory suggest that *Tet1* expression is low in the epiblast and EpiSCs in much the same way as many of these early responders to loss of pluripotency (T. A. Hore *et al*. 2012, unpublished data). Thus, it could be that loss of *Tet1* expression during implantation is important for the methylation of these genes in the epiblast, and their subsequent stable silencing in somatic tissues thereafter.

In the same way that silencing *Tet1* may be important for differentiation, upregulation of *Tet1* may be critical for reprogramming epiblast cells into pluripotent PGCs *in vivo*, or EpiSCs into iPS cells *ex vivo*. Indeed, induction of *Tet1* occurs during iPS reprogramming of fibroblasts [101], suggesting that it may play a role in the reprogramming process, potentially via DNA demethylation. There is precedent for such a hypothesis: firstly, inhibition of DNMT1, and treatment with 5-azacytidine both have the effect of enhancing reprogramming of fibroblasts into an ES-cell-like state [102]. Moreover, the cytosine deaminase AID is also likely to contribute to reprogramming—knockdown of AID in heterokaryons formed by fusing a differentiated cell with an ES cell effectively abolishes the ability of the ES cell to activate the pluripotency network of the differentiated cell [31]. Extrapolating from these results, it seems possible that in addition to AID and TET1, members of the BER pathway may also contribute to the enhancement of artificial reprogramming via DNA demethylation. Of particular interest in this regard is the thymine glycosylase TDG, which appears to connect the oxidation pathway with BER. However, despite these tantalizing possibilities, it must be stressed that there is still much work that needs to be done. In particular, none of the possible DNA demethylating proteins have actually demonstrated enhancement of reprogramming in systems that could be applied to a biotechnological or clinical setting. Moreover, the level of imprecise DNA demethylation imparted by protein overexpression has yet to be quantified, and could have serious implications for the ability of iPSCs to function properly following subsequent differentiation.

## 5. DNA methylation: a vital regulator of the mammalian life cycle?

Mammalian embryonic development is an incredibly complex undertaking that requires an extensive capacity for plasticity to allow for the drastic changes in cell fate and developmental potential. In this review, we have highlighted the alternating phases of DNA methylation erasure and re-establishment during mammalian development that reflect these developmental changes (figure 1). It is widely accepted that epigenetic reprogramming in mammalian development is required for resetting imprints for the next generation; however, one of the most intriguing and relevant questions that remains unanswered is whether the reprogramming of DNA methylation outside imprinted genes also plays a crucial role in mammalian development.

This question is difficult to address, as a comprehensive understanding of the mechanisms involved remains to be achieved; emerging evidence indicates that this picture is additionally complicated by functional redundancy. Nevertheless, knockout studies have yielded some insight. In PGCs, depletion of *Aid* impairs global methylation erasure but—notably—does not restrict fertility [7]. The presence of alternative demethylation pathways may prevent a more significant impact on methylation levels, particularly at the crucial imprinted regions, which could explain the viability of the resultant germ cells. The demethylation of the paternal pronucleus in the zygote was linked to developmental viability when it was shown that blocking the oxidative demethylation pathway by genetic inactivation of TET3 causes partial embryonic lethality [9]. A lack of demethylation at the *Nanog* and *Oct4* promoters and subsequent impaired activation in the early embryo may have contributed to this phenotype. It is also possible that development may have been affected by the absence of oxidized cytosine marks (5hmC, 5fC and 5caC) usually retained into early cleavage divisions (see above), rather than the abrogation of 5mC removal *per se*. Furthermore, while global paternal demethylation is a feature of many mammalian zygotes, its extent is variable, and in some species it is followed by significant remethylation [103–107]. In mouse, fertilization of oocytes with round spermatids results in partial demethylation that is reversed by remethylation before metaphase; this methylated paternal genome does not preclude normal embryonic development [108,109]. These studies indicate that hypomethylation of the paternal genome at the end of the first cell cycle may not be an absolute requirement for embryogenesis. Thus, conclusive evidence that DNA methylation reprogramming is essential for normal development is still lacking; however, we anticipate that advances in our understanding of the molecular processes involved, together with the ability to overcome redundancy through the simultaneous targeting of multiple pathways, will allow researchers to address this fundamental question in the near future.

So, what functional purpose does the global erasure of DNA methylation marks in PGCs and in the zygote serve? To date, epigenetic characteristics with transgenerational inheritance in mammals have been difficult to find, and these effects are often modest [7,110] or involve IAPs, which are particularly resistant to epigenetic modification [111]. Thus, it seems likely that such global reprogramming provides a safety net for the correction of epimutations at the generational boundary. In line with this idea, plants do not undergo global DNA demethylation in the germline [70], and appear to have a greater capacity for transgenerational epigenetic inheritance [112].

Beyond this, reprogramming occurs at significant transitions in the developmental programme of the cell—this may necessitate a ‘resetting’ of the epigenome in order to provide a blank canvas on which to paint new epigenetic marks for the totipotent state and subsequent lineage decisions (in the zygote), and the creation of germ cell identity (in PGCs). Furthermore, global removal of methylation marks may be a prerequisite for the large-scale transcriptional changes that occur at these time points: in PGCs, the somatic cell programme becomes silenced and the germ cell programme activated [16], while zygotic genome activation at the two-cell stage represents a major transition from transcriptional quiescence at fertilization [113]. The same concept can be applied to experimental reprogramming, which is significantly enhanced by demethylating agents and DNA methyltransferase inhibitors [97,102,114]. It is not that removal of DNA methylation marks is responsible for activation of the pluripotency network in artificially reprogrammed cells—clearly, this is due to the action of key transcription factors such as *Oct4* and *Nanog*. However, a demethylated state may increase epigenomic plasticity to facilitate the enormous transcriptional changes associated with the erasure of somatic cell fate and re-establishment of pluripotency.

While reprogramming can be linked to widespread changes in gene expression, it is important to note that much of the methylation loss *in vivo* occurs at repetitive sequences, particularly in the zygote [7,13,60]. Is this merely a consequence of the need for global rather than targeted reprogramming? This seems unlikely, as the activation of Line1 and some LTR repetitive elements is potentially required for progression beyond the four-cell stage [108,115]. Transposable elements can influence transcription at neighbouring loci by a variety of means [116]; thus this activity may be intricately linked to the transcriptional programme of the early embryo, and possibly also in PGCs. Alternatively, as in plants, transposable elements may be relieved from transcriptional repression in order to expose them to the piRNA machinery, leading to re-establishment of silencing [116–118]. This must be balanced with the danger of uncontrolled transposition in the genome and may explain why the process of methylation erasure from repetitive elements is incomplete. In both the zygote and PGCs, certain repetitive elements such as IAPs escape demethylation [13,14]; in PGCs, this has been shown to also protect adjacent sequences from reprogramming [15]. The mechanism by which IAPs resist DNA methylation is currently unknown. One possibility is that IAPs are less prone to active removal of DNA methylation compared with sequences that lose methylation. Alternatively, it is possible that they are better enabled to maintain DNA methylation, perhaps through more efficient targeting of DNMT1 or are for some reason more susceptible to de novo methylation. This latter point may relate to recent evidence suggesting that genes involved in the piRNA pathway become transcriptionally activated in PGCs upon promoter demethylation [119]. It is thought that this may provide an elegant sensory mechanism that couples global methylation erasure, activation of the piRNA machinery and repeat silencing.

The re-establishment of pluripotency is a feature strongly associated with epigenetic reprogramming, which includes demethylation of promoters of pluripotency factors and their transcriptional activation. Indeed, pluripotency markers are expressed in the early embryo and in PGCs (see above). However, the causal relationship between the two is unclear. Is it DNA methylation erasure that activates the pluripotency network or is the latter activated by other stimuli and then in turn induces epigenetic reprogramming?

There are many exciting questions to be addressed in epigenetic reprogramming and we are now only beginning to understand the molecular mechanisms involved. This understanding, the establishment of *in vitro* systems and the rapid development of new technologies will hopefully allow researchers to answer these key questions in the near future.
